# Evidence-based guidelines for the use of biochemical markers of bone turnover in the selection and monitoring of bisphosphonate treatment in osteoporosis: a consensus document of the Belgian Bone Club

**DOI:** 10.1111/j.1742-1241.2008.01911.x

**Published:** 2009-01

**Authors:** P Bergmann, J-J Body, S Boonen, Y Boutsen, J-P Devogelaer, S Goemaere, J-M Kaufman, J-Y Reginster, V Gangji, Members of the Advisory Board on Bone Markers

**Affiliations:** 1 Laboratory of Clinical Chemistry, CHU Brugmann, Université Libre de BruxellesBruxelles, Belgium; 2 Department of Medicine, CHU Brugmann, Université Libre de BruxellesBruxelles, Belgium; 3 Center for Metabolic Bone Diseases, KU-LeuvenLeuven, Belgium; 4 Department of Rheumatology, Mont-Godinne University Hospital, UCL-BrusselsBrussels, Belgium; 5 Arthritis Unit, Saint-Luc University Hospital, UCL-BrusselsBrussels, Belgium; 6 Department of Endocrinology, University Hospital, UGentGent, Belgium; 7 Bone and Cartilage Metabolism Research Unit, Department of Public Health, Epidemiology and Health Economics, ULg-LiègeLiège, Belgium; 8 Department of Rheumatology and Physical medicine and Rehabilitation, Erasme Hospital, Université Libre de BruxellesBruxelles, Belgium

## Abstract

**Objectives:**

To review the clinical value of bone turnover markers (BTM), to initiate and/or monitor anti-resorptive treatment for osteoporosis compared with bone mineral density (BMD) and to evaluate suitable BTM and changes in BTM levels for significance of treatment efficiency.

**Methodology:**

Consensus meeting generating guidelines for clinical practice after review and discussion of the randomised controlled trials or meta-analyses on the management of osteoporosis in postmenopausal women.

**Results:**

Although the correlation between BMD and BTM is statistically significant, BTM cannot be used as predictive markers of BMD in an individual patient. Both are independent predictors of fracture risk, but BTM can only be used as an additional risk factor in the decision to treat. Current data do not support the use of BTM to select the optimal treatment. However, they can be used to monitor treatment efficiency before BMD changes can be evaluated. Early changes in BTM can be used to measure the clinical efficacy of an anti-resorptive treatment and to reinforce patient compliance.

**Discussion:**

Determining a threshold of BTM reflecting an optimal long-term effect is not obvious. The objective should be the return to the premenopausal range and/or a decrease at least equal to the least significant change (30%). Preanalytical and analytical variability of BTM is an important limitation to their use. Serum C-terminal cross-linked telopeptide of type I collagen (CTX), procollagen 1 N terminal extension peptide and bone specific alkaline phosphatase (BSALP) appear to be the most suitable.

**Conclusion:**

Consensus regarding the use of BTM resulted in guidelines for clinical practice. BMD determines the indication to treat osteoporosis. BTM reflect treatment efficiency and can be used to motivate patients to persist with their medication.

**Review Criteria**

An extensive Pubmed search was used to identify the relevant literature, which included randomised controlled trials and meta-analyses, considering the use of biochemical markers of bone turnover in osteoporosis. A critical appraisal of the data was obtained through consensus expert meetings. The guidelines for the clinical practice are the conclusions of these analyses and discussions.

**Message for the Clinic**

Standardised guidelines defined in the consensus ‘how to use bone turnover markers’ will help clinicians in a better management of osteoporosis. As bone turnover markers decrease rapidly after initiation of anti-resorptive treatment, they represent useful surrogate markers not only to reflect therapeutic success but also to monitor patient’s compliance.

Osteoporosis is a major health issue. It is a disease characterised by low bone mass and altered bone architecture leading to an increased susceptibility to fractures (1).Osteoporosis is defined by a value of bone mineral density (BMD), measured by dual-energy X-ray absorptiometry (DXA) on the spine or hip, more than 2.5 SD below the normal peak values for young adults (*T*-score < −2.5) (WHO criteria) or by the occurrence of a low trauma fracture.

So, BMD measurement is the pivotal mainstay in the decision to initiate an anti-resorptive treatment. BMD is used also to monitor treatment efficacy. However, BMD alone presents some shortcomings, both for the diagnosis of osteoporosis and for the treatment monitoring. With regard to diagnosis, many fractures occur in patients who do not have a *T*-score < −2.5. As far as monitoring is concerned, BMD changes in response to anti-resorptive treatment are slow (2–5% per year, or a maximum of < 3% in 3–6 months). So, at least 1 year of treatment is necessary before a significant change in BMD can be observed and, furthermore, absence of BMD increase does not imply absence of therapeutic response. The changes in bone turnover rate are much faster and several analytes, which can be measured easily in serum or urine, reflect the rate of bone formation or bone resorption. These bone turnover markers (BTM) will be presented here. We shall review their suitability and potential added value in the selection of patients to be treated and in the follow-up of patients undergoing treatment for osteoporosis.

## Methodology

We included randomised controlled trials and meta-analyses in postmenopausal women, comparing interventions currently registered in Belgium for the management of osteoporosis with placebo. The results had to be reported with a follow-up of at least 1 year. The relevant articles of the literature review were discussed and a critical appraisal of the data was obtained through a consensus experts meeting.

The guidelines for clinical practice, phrased as ‘Consensus of the Belgian Bone Club’ (Table 1), are the conversion of the conclusions of the consensus expert meeting into daily practice, reviewed by the members of the Advisory Board on Bone Markers.

**Table 1:** Consensus of the Belgian Bone Club regarding the use of bone turnover markers: in practice

	Indication for anti-resorptive therapy:BMD hip/spine: *T*-score less than −2.5 SDor low trauma fracture
	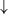
	Assess baseline BTM levels: fasting serumBSALP or CTX or P1NP
	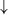
Day 0	Start bisphosphonate therapy
3 months	Control BTM levels: decrease ≥ 30%
	If not:
	Check compliance
	Check if drug is taken properly (for instance,not with milk or calcium supplement,or waters rich in calcium)
	Drug storage
6 months	Control BTM levels: decrease > 30%
	+ return to premenopausal state
1 year	Reassess BTM

BMD, bone mineral density; BTM, bone turnover markers; BSALP, bone-specific alkaline phosphatase; P1NP, procollagen 1 N terminal extension peptide.

## Biomarkers of bone turnover

To predict bone loss, we should measure at one time-point the rate of bone turnover and the balance between formation and resorption. But, by measuring the concentration of BTM (corresponding to the ratio of their production and degradation), we cannot assess quantitatively the amount of matrix deposited and mineralised or destroyed per unit of time, even though some BTM are more linked to bone resorption and others to bone formation.

### Bone formation

#### Bone-specific alkaline phosphatase

There are several isoforms of alkaline phosphatase originating from many tissues, mainly liver and bone, with bone contributing for 40–50% in normal adults. The bone enzyme can be separated from the other forms by chemical separative methods such as lectin precipitation, heat resistance or electrophoresis (2). Automation of the specific immunoassays for bone-specific alkaline phosphatase (BSALP) has improved the analytical reproducibility to < 5%. Unfortunately, there is a significant cross-reactivity (± 15%) with the liver form (3), which can be clinically relevant when the patient suffers from liver disease. The half-life of BSALP is 1–2 days, making it less sensitive to circadian variation than other markers with a shorter half-life. The long-term intra-individual variability of BSALP is 10%, and this biological variability represents the major component of variability since the improvement of analytical methods.

#### Osteocalcin

Osteocalcin is a small protein synthesised exclusively by osteoblasts (and odontoblasts). It is deposited into the bone matrix to form the major non-collagenic part and can be released in part into the circulation. The flux of osteocalcin towards the serum also results from matrix resorption, thus it is not a pure osteoblast function marker. Osteocalcin has a circadian rhythm and is higher in the early morning (4). It is excreted by glomerular filtration and its concentration is increased when glomerular filtration decreases (5).

Osteocalcin can be measured by several immunoassays, but its measurement is complicated by the presence in variable amount of several fragments, by a lack of uniform standardisation (6) and by its degradation in the serum even in the absence of haemolysis causing an important preanalytical problem. The conservation problem can be improved, but only in part, by using an immunoassay, which recognises a large N-terminal fragment (7).

#### Procollagen I extension peptides

Type I collagen is synthesised as a precursor flanked at its C- and N-termini with extension peptides, which are cleaved when the collagen is deposited to form the bone matrix. The catabolism of both extension peptides, procollagen 1 C terminal extension peptide and procollagen 1 N terminal extension peptide (P1NP) is under hormonal control, but their concentration is not dependent of renal function. As bone is remodelled faster than other conjunctive tissues, its contribution to serum extension peptides is dominant, at least in the absence of any conjunctive disease. Both peptides can be measured by immunoassay (8,9) and have been shown to follow the expected variations in bone turnover in different physiological and pathological conditions. P1NP circulates as a trimer, which is rapidly degraded at 37°; recognition of the monomer varies between assays (10). They follow a circadian rhythm and are higher in early morning. The intra-individual coefficient of variation (CV) is 12.4% for P1NP; combining analytical and biological variability revealed a critical difference between two successive serial measurements of 38% (11).

### Bone resorption

#### Acid phosphatase

Osteoclasts produce an acid phosphatase isoenzyme, which is not inhibited by tartrate [type 5 tartrate resistant acid phosphatase (TRAP)]. Total TRAP, measured by chemical methods, has long been considered as a marker of bone resorption. However, total TRAP is influenced by enzymes originating from the erythrocytes and platelets, and its measurement can be hampered by circulating inhibitors. Now it can be measured in serum by immunoassays using an immunometric format with a precision of 5% (mass measurement) or 15% (enzyme activity of the captured protein). A kinetic assay to measure specifically type 5b TRAP, a desialylated isoenzyme present only in osteoclasts and alveolar macrophages, has also been described, with a CV of 5–10%. Increased type 5 TRAP levels have been described in diseases characterised by increased bone resorption, such as primary or secondary hyperparathyroidism, Paget’s bone disease or metastatic bone disease. There are few studies on type 5b TRAP in osteoporosis studies.

#### Pyridinoline and deoxypyridinoline crosslinks

These crosslinks (aldehyde links between lysine or hydroxylysine residues) are formed between collagen molecules and they stabilise the conjunctive tissue. They are released into the circulation and excreted into the urine when collagen is catabolised. They reflect only collagen degradation. Deoxypyridinoline (DPD) is found only in skeletal tissue, but both crosslinks mainly originate from bone resorption. When bone metabolism is normal, 50% of the crosslinks are free and 50% bound to peptides (12). The measurement most representative of true bone resorption is probably that of the total crosslinks. It was shown indeed that when high turnover bone diseases were treated with anti-resorptive drugs, there was only a minimal decrease in the free crosslinks, while the peptidic forms decreased dramatically with total crosslinks in between (13). Today most studies are based on immunoassay measurements, mainly of the peptidic forms. They follow a circadian rhythm and are higher in the early morning.

#### Telopeptides of type I collagen

These peptides are the non-helical region of type 1 collagen where the crosslinks attach. The measured molecules are either a trimeric carboxyterminal telopeptide (ICTP), which is measured in serum by radioimmunoassay (14) ICTP or a synthetic peptide sequence containing the crosslink site which can be measured in serum or urine [C-terminal crosslinked telopeptide of type I collagen (CTX)] (15). There are four isomers of CTX, according to the isomerisation of the aspartate (native α- and transformed β-CTX) and to its racemisation (l or d). Both racemisation and isomerisation increase with tissue age; thus measurement of the different forms could give an insight into the mean age of bone tissue (with α/β higher if bone turnover is increased). Practically, there are competition assays, which measure the two isomers in the urine and β-CTX can be measured in the serum with a sandwich immunoassay. Serum and urine CTX values are highly correlated. Another assay called NTX recognises an epitope of the N-terminal telopeptide of the α-2 chain of collagen I (16). They follow a circadian rhythm and are higher in the early morning.

## Prediction of postmenopausal bone loss using BTM

Postmenopausal oestrogen deficiency causes an acceleration of bone remodelling. As osteoclasts, responsible for bone resorption, are triggered by oestrogen deficiency, there is an imbalance in bone formation and bone resorption leading to bone loss. The loss resulting from this imbalance is faster in the trabecular than in the cortical compartment of bone (17,18). The increase in the parameters of bone resorption varies from 0% to 150%, with a subsequent increase of 0% to 100% in markers of bone formation (12,19). The increased remodelling can persist until late in life (19–21), more than 30 years postmenopause.

Various longitudinal studies strongly support the fact that high bone turnover leads to faster bone loss than normal or low bone turnover (20,22–25). In one study, using markers such as total serum alkaline phosphatase, fasting urinary calcium and hydroxyproline, women diagnosed as fast losers, based on markers, had lost 26.6% of bone mass after 12 years compared with 16.6% in those classified as slow losers (23). In elderly patients, aged 75 years, significant associations were found between serum osteocalcin, serum CTX, TRAP and urinary DPD and the areal BMD of the leg region, derived from the whole body measurement (26). No correlation was found with the areal BMD of the spine. It is well known that degenerative osteoarthritic changes at the spine can be a confounding factor for BMD measurements by DXA. The progressive availability of BTM with a better specificity and precision should increase their capability to predict the rate of bone loss.

Although the correlation between BMD and the levels of BTM is statistically significant (27,28), it is not strong enough to use BTM as predictive markers of BMD in an individual patient.

## Fracture risk assessment: BTM is an independent risk factor

The major complication of osteoporosis is the occurrence of fractures for minimal trauma with their inherent increase in morbidity and mortality. A decrease in one *T*-score in BMD, measured by DXA, is associated with a doubling of the relative risk of fracture at the spine, hip and forearm (29).

Several studies have shown that some markers of bone resorption are independent predictors of fractures, especially spine and hip (30–35). In osteopenic women, the 10-year probability of fracture amounted to 26% if at least one risk factor was present (amongst age, elevated BSALP and prior fracture) vs. 6% only in women without any of the three risk factors (34). Elevated serum CTX levels were associated with a significantly increased risk of osteoporotic fractures (32,35), but only above a certain threshold, suggesting that an increased resorption together with an increased activation frequency (=the appearance rate of the basic multi-cellular unit – BMU – in a histology slide) could lead to a more pronounced fragility of bone superimposed to the fragility already induced by a low BMD (21).

There was also an association between the elevation of markers of bone formation and the risk of fracture (32–34). An increased risk of hip fracture was observed in ambulatory and institutionalised elderly women with low levels of undercarboxylated osteocalcin, an index for a low vitamin K status (36,37).

Bone turnover markers and BMD predict fracture risk independently. When both factors are altered, the fracture risk is more increased than for each of them considered separately. In the OFELY study, women suffering from osteodensitometric osteoporosis of the hip combined with an elevated serum CTX had a relative risk of fractures within 5 years amounting to 55%. This is significantly higher than the relative risk linked to an isolated low BMD (39%) or an isolated elevated CTX (25%) (32). It must be mentioned, however, that the markers can only be used as an additional risk factor, not as a surrogate for BMD measurement in the decision to treat.

## Selection of a specific treatment

Theoretically, a better response to anti-resorptive treatment should be expected in patients with a high bone turnover rate. However, current data do not support the use of BTM to select the optimal treatment, as the relationship between BTM concentration and response to anti-resorptive treatment is, at best, weak (38–41).

In a *post hoc* analysis of the Fracture Intervention Trial (FIT), there was no relationship between pretreatment P1NP, BSALP or serum C-terminal crosslinked telopeptide of type I collagen (sCTX) and alendronate efficacy for incident spine fractures among osteoporotic women (39). Nevertheless, a recent pharmacoeconomic study (Markov model) concluded that measurement of BTM has the potential to identify a subset of postmenopausal women (top BTM quartile), without osteoporosis by BMD criteria, for whom alendronate therapy to prevent fracture is cost-effective [costs per quality adjusted life years (QALY) gained at 34,000 and 50,000 USD for women aged 70 years with high bone turnover and femoral neck BMD *T*-score of −2.0 and −1.5 respectively] (38).

A similar analysis of the risedronate phase III clinical programme showed that the reduction in the incidence of vertebral fractures was independent of baseline urinary DPD. However, the number needed-to-treat (NNT) to avoid one vertebral fracture at 12 months was 15 in the group of patients with high DPD and 25 in patients with low DPD, an observation which is not unexpected, based on the influence of the prevalent absolute risk on NNT calculation (40). The authors concluded that although the reduction in overall fracture risk seems to occur independent of baseline bone turnover, patients’ stratification by pretreatment bone resorption rate seems to make some sense from a pharmacoeconomic point of view (40,41).

## Early changes of BTM, changes of BMD and fracture risk

In the Early Postmenopausal Intervention Cohort study, early postmenopausal women, receiving alendronate for the prevention of osteoporosis, with a decrease of 40% for urinary N-terminal telopeptide of type 1 collagen (uNTX) or 20% for osteocalcin at month 6 had a 92% probability of a 2-year positive response in spine BMD. In contrast, the poor specificity and negative predictive value of these percentual cut-offs of BTM changes implied that a change in uNTX or osteocalcin above the cut-point was a poor predictor of bone loss during alendronate treatment (42).

In a smaller cohort of French osteoporotic women, the authors claimed that after 4 months of alendronate, sCTX and to a lesser extend uNTX, were the best predictors of a significant gain in spine BMD after 1 year of therapy (43).The authors from the FIT concluded that the correlation between early reduction in bone turnover and long-term fracture reduction during alendronate treatment was at least as strong as that observed with 1-year changes in BMD. For patients with a decrease of 30% in bone turnover, compared with those with a decrease of < 30%, the risk of experiencing a fracture at the end of the trial was significantly lower for spine and hip (44).

In the risedronate vertebral fracture trial, the relationship between vertebral fracture risk and changes from baseline in sCTX and uNTX were not linear (p < 0.05). There was little further improvement in fracture benefit below a decrease of 55–60% for sCTX and 35–40% for uNTX. The authors concluded that the decrease in bone resorption in patients taking risedronate accounts for a large proportion of the reduction in fracture risk but that there may be a level of bone resorption reduction below which there is no further fracture benefit (45).

With continuous (2.5 mg daily) or intermittent (20 mg every other day for 12 doses every 3 months) oral doses ibandronate which were linked to a significant reduction in spinal fractures, similar to that seen with alendronate or risedronate, the rate of bone turnover was reduced by 50–60%, a magnitude also within the range observed with the efficacious oral bisphosphonates (46). For further clinical development of ibandronate, even the role of BTM as a predictor of efficacy was emphasised. Actually, a pharmacokinetic-pharmacodynamic model, accurately describing the urinary excretion of CTX was used to select the appropriate once-monthly dose of ibandronate (47).Clinical studies showing the non-inferiority and/or superiority of the 150 mg monthly oral regimen on BMD, over the daily 2.5 mg dose, the dose which previously demonstrated anti-fracture efficacy (48), confirmed *a posteriori* the interest of a pharmacostatistical model based on BTM changes to predict the effect of a particular dosage of ibandronate on hard end-points (49).

Data from randomised clinical studies of ibandronate, given orally or intravenously, in different doses and for variable time intervals to women with osteoporosis, were examined to explore the relationship between intermittent bisphosphonate therapy, changes in bone resorption and fracture risk. The magnitude of the reduction in the rate of bone resorption at the end of the drug-free interval, rather than its fluctuation pattern after bisphosphonate administration, determines anti-fracture efficacy, provided that these fluctuations occur within the premenopausal range (50).

## Monitoring bisphosphonate treatment

Bisphosphonate therapy inhibits bone resorption and decreases the rate of bone remodelling. So, BTM can be used to measure the effect of an anti-resorptive treatment. However, determining a threshold of BTM to attain an optimal long-term effect is not obvious.

### Setting objectives

As opposed to the WHO definition of osteoporosis on basis of a low BMD, currently unanimously accepted criteria to categorise low or high bone turnover, compared with premenopausal state, are not available. Furthermore, as for BMD, there is some overlap between healthy pre- and postmenopausal women, as well as between osteopenic and osteoporotic populations in the values of bone remodelling parameters (51).

The objective should be the return of BTM levels to the premenopausal range. However, half of the women with osteoporosis have BTM levels within the premenopausal range. In these patients, the goal should be a decrease at least equal to the least significant change (LSC). It is interesting to underline that changes in BMD do not explain alone the anti-fracture efficiency of treatment: some patients with unchanged or even decreased BMD may be protected against fracture. Thus, as for determining fracture risk, BTM could be complementary to BMD to predict the anti-fracture efficacy.

### Adherence and persistence

In a study testing the impact of monitoring on adherence and persistence with anti-resorptive treatment, physician’s reinforcement on adherence to bisphosphonate treatment, using BTM resulted in a lower incidence of new radiologically determined vertebral fractures (odds ratio 0.4; 95% CI: 0.2–1.0). The positive impact of a positive feedback to the patient (by telling him his BTM levels were decreased) was only seen when the decrease in BTM levels was marked (> 30% decrease) (52).

In a randomised controlled trial, monitoring on adherence to and persistence with anti-resorptive treatment were performed by nursing staff. The use of BTM and the supplying of complementary information on the importance of compliance for treatment response were compared with no monitoring. In the monitored group, cumulative adherence to therapy was increased by 57% (p = 0.04), with a trend to persist with therapy for 25% longer (p = 0.07), both compared with no monitoring. However, feedback on BTM alone or nurse-monitoring alone did not show any difference in adherence or persistence rates. Nevertheless, adherence at 1 year was correlated with BMD increase (hip; r = 0.28; p = 0.01) and percentage change in uNTX (r = −0.36; p = 0.002) as response to treatment efficacy (53). We should also be aware that a negative feedback (by telling there is no reduction in BTM) could stimulate the patient in his non-compliance.

## Limitations of BTM use: variability

The production of BTM depends not only on the rate of bone turnover, but also on the skeletal size, reflecting mainly trabecular bone turnover, which is 4 to 5 times more active than cortical bone. A localised bone disease, bed rest and fracture healing can interfere with values. For urine markers, the expression as a ratio to creatinine introduces another factor of variability.

The relationship between the measured concentration and what happens in the bone differs intra- and inter-individually. Inter-individual variability is largely determined by differences in age, gender and menopausal status.

### Analytical variability

With the improvement in analytical methods, particularly with the introduction of automated immunoassays, the analytical CV remains around 5%. The absence of uniform standardisation still is a concern and makes it difficult to compare values obtained by different methods in different laboratories (54). That is the reason why all measurements for one individual should be done in the same laboratory. So, the main source of variability is preanalytical, mostly sample conservation and biological variability.

### Conservation variability

Concerning sample conservation, osteocalcin and acid phosphatase are the most difficult to handle. Collagen peptides and alkaline phosphatase are much more resistant to degradation. Pyridinoline crosslinks are light sensitive and degraded under the influence of intense UV irradiation.

### Biological variability

In adults, the main source of undesirable but controllable biological variability is the circadian rhythm, with higher values in the early morning hours, then a steep decrease in the morning, to attain a nadir at the end of the afternoon (55,56). Most markers follow the same pattern, with the exception of alkaline phosphatase because of its longer half-life. The steepest decline during the morning has been described for serum CTX, but it is attenuated if the patients remain in the fasting state. Practically, it implies that the sampling time for measuring bone markers has to be strictly controlled. There is also a seasonal variation in bone turnover, due in part to fluctuations of vitamin D repletion.

### Serum vs. urine markers

Many studies have shown that the intra-individual variability is around 10% for serum markers and between 15% and 25% for urine markers. So, the signal to noise ratio is better for serum markers. This has important consequences for follow up. Indeed, if a marker is measured once, the confidence interval of the result is ±1.96 × SD. The LSC, defined as a difference reflecting a real change with a 5% chance of type 1 error (false-positive), is a change surpassing 2.8 × CV. Thus, for a CV of 10%, a change of at least 25–30% must be observed for serum markers to consider that there is a significant evolution, while a change of 40–70% is required for urine markers. Fortunately, decreases in this order of magnitude are observed in individual patients with anti-resorptive therapy. However, one must be aware of the risk of type 2 error (false-negative), and it must be avoided to change treatment on the basis of an insufficient evolution, at least on two sequential measurements only.

## Consensus of the Belgian Bone Club

### Choice of markers

- Serum markers do not need correction for glomerular filtration rate; automated technology for measurement available for serum CTX and serum P1NP.
- Osteocalcin not optimal in routine clinical practice because of its fragility.
- Serum BSAP can be added in patients without liver problems (less sensitive to circadian rhythm).
- Measurements for one individual must be performed in the same lab using standard procedures; samples should be taken while fasting and always at the same time of day.
- There are no data to determine the optimal postintake period to take a blood sample for follow-up of the markers, but at least 7 days after the intake seems to have some support, understanding that it is always the same period in one individual patient.

### Use of markers (pretreatment)

- Baseline BTM levels cannot be the base of anti-resorptive drug choice, nor do they predict treatment effect.
- Bone turnover markers are an independent risk factor of fracture.

### Use of markers (follow-up)

- This use relies on the definition of LSC (around 30% for serum markers and 50–60% for urine markers).
- Only a decrease higher than the LSC can be interpreted as showing a biological effect.
- Early changes in BTM (baseline vs. posttreatment) show a biological effect of the medication, proving patient compliance and persistence.
- The early decrease in BTM level is probably predictive of bone gain and anti-fracture efficacy. However, it is not known to which level BTM should be decreased to optimise anti-fracture efficacy.
- As we cannot link BTM decrease to a threshold, using premenopausal range seems a rational objective to achieve.
  1. The levels of osteoporotic patients whose BTM levels are still in premenopausal range must still decrease by 30%.
  2. Premenopausal ranges have been well defined.
  3. Analytical differences must be resolved by using the same lab for one individual patient.
